# Effects of dodecacalcium hepta-aluminate content on the setting time, compressive strength, alkalinity, and cytocompatibility of tricalcium silicate cement

**DOI:** 10.1590/1678-7757-2018-0247

**Published:** 2019-01-07

**Authors:** Yoorina Choi, Jong-Lye Bae, Hee-Jin Kim, Mi-Kyung Yu, Kwang-Won Lee, Kyung-San Min

**Affiliations:** 1 Wonkwang University Dental Hospital, Department of Conservative Dentistry, Iksan, Korea; 2 Chonbuk National University, School of Dentistry and Institute of Oral Bioscience, Department of Conservative Dentistry, Jeonju, Korea; 3 Kosin University, College of Medicine, Department of Dentistry, Busan, Korea; 4 Chonbuk National University, Research Institute of Clinical Medicine, Jeonju, Korea; 5 Chonbuk National University Hospital, Biomedical Research Institute, Jeonju, Korea

**Keywords:** Calcium silicate, Dental pulp, Physicochemical analysis, Material testing

## Abstract

**Objective:**

This study aimed to investigate the effects of dodecacalcium hepta-aluminate (C12A7) content on some physicochemical properties and cytocompatibility of tricalcium silicate (C3S) cement using human dental pulp cells (hDPCs).

**Material and Methods:**

High purity C3S cement was manufactured by a solid phase method. C12A7 was mixed with the cement in proportions of 0, 5, 8, and 10 wt% (C12A7-0, −5, −8, and −10, respectively). Physicochemical properties including initial setting time, compressive strength, and alkalinity were evaluated. Cytocompatibility was assessed with cell viability tests and cell number counts. Statistical analysis was performed by using one-way analysis of variance (ANOVA) and Tukey's test (p<0.05).

**Results:**

The initial setting time of C3S-based cement was shorter in the presence of C12A7 (p<0.05). After 1 day, C12A7-5 showed significantly higher compressive strength than the other groups (p<0.05). After 7 days, the compressive strength of C12A7-5 was similar to that of C12A7-0, whereas other groups showed strength lower than C12A7-0. The pH values of all tested groups showed no significant differences after 1 day (p>0.05). The C12A7-5 group showed similar cell viability to the C12A7-0 group (p>0.05), while the other experimental groups showed lower values compared to C12A7-0 group (p<0.05). The number of cells grown on the C12A7-5 specimen was higher than that on C12A7-8 and −10 (p<0.05).

**Conclusions:**

The addition of C12A7 to C3S cement at a proportion of 5% resulted in rapid initial setting time and higher compressive strength with no adverse effects on cytocompatibility.

## Introduction

Since its introduction in the 1990s, mineral trioxide aggregate (MTA) has been widely used in the endodontic field for purposes including retrograde filling, perforation repair, apexification, and vital pulp therapy.^1^ Considerable evidence has demonstrated the excellent biocompatibility and sealing ability of MTA and promising outcomes for endodontic procedures.^2^ ^,^ ^3^ Nevertheless, because of drawbacks including long setting time and initial wash-out possibility, poor handling characteristics, discoloration of teeth, and heavy metal content, the development of novel MTA-like calcium silicate (CS) cement to overcome these drawbacks and improve biocompatibility and clinical convenience of MTA is an important goal.^4^

The composition of MTA is similar to that of Portland cement except for the presence of radiopacifier like bismuth oxide, which is generally composed of tricalcium silicate (C3S), dicalcium silicate (C2S), tricalcium aluminates (C3A), tetracalcium aluminoferrite, and other ingredients.^5^ Among these, C3S is one of the major constituents of MTA.^6^ C3S forms into calcium silicate hydrates (C-S-H) through hydration reactions with water, which contribute to the spontaneous development of strength.^7^ Despite its excellent *in vitro* bioactivity and biocompatibility,^8^ C3S alone has limitations in clinical contexts due to its long setting time and low mechanical strength during the early stages of hydration.^9^

Tricalcium aluminate (C3A) is the most reactive part of Portland cement.^10^ When C3A is mixed with calcium silicate, it contributes to the initial hydration process of the cement.^10^ In studies, C3A mixed with calcium silicate cement showed a faster hydration rate and higher initial mechanical strength than non-mixed cement.^7^ ^,^ ^9^ ^,^ ^10^ Dodecacalcium hepta-aluminate (C12A7), one of the stable phases of calcium aluminates (CA), is also expected to react rapidly with water and may provide beneficial properties during the early hydration stages of calcium silicate. However, there have been no attempts to mix C12A7 in C3S cement and evaluate the effects of C12A7 on cement in dental contexts. This study aimed to evaluate the effects of C12A7 on C3S cement regarding some physicochemical properties and cytocompatibility with hDPCs. We manufactured high-purity cement with uniformly fine particles to determine the optimal component ratio of C3S/C12A7 for use as an endodontic biomaterial. The null hypothesis was that cements with different C12A7 contents would not significantly differ in practical properties.

## Material and methods

### Material preparation

For the manufacture of C3S cement powder, calcium carbonate (Sigma-Aldrich, St. Louis, MO, USA) and silicon dioxide (Junsei Chemical, Kyoto, Japan) were uniformly mixed in a 3:1 molar ratio and stirred by ball milling for 1 h in ethanol. The powder (10 g) was put into a mold 15-mm in diameter and pressed uniaxially at pressures of 2 to 3 t. After the cylinder-type pelletized samples were desiccated at 40°C for 12 h, the sample was heated at a rate of 10°C/min to the sintering temperature of 1400-1500°C, held for 1 to 20 h, and then cooled. Sintering process was repeated four times to achieve higher proportions of C3S in the cement. The sintered material was ground to powders less than 8 μm in diameter by using air jet mill (CGS16, NETZSCH GmbH, Selb, Germany) with 6000 rpm. The characteristics of prepared C3S cement powder were evaluated with X-ray diffraction (XRD) analysis (D/MAX 2500V/PC, Rigaku, Tokyo, Japan), a laser diffraction particle size analyzer (LS 13 320, Beckman Coulter Inc., Miami, FL, USA), and a scanning electron microscope (SEM; S-4300, Hitachi, Tokyo, Japan). C12A7 was manufactured as follows; calcium oxide (Sigma-Aldrich) and aluminum hydroxide (Duksan pure chemical, Ansan, Korea) were uniformly mixed at a 12:7 molar ratio. The mixed compounds were heated at 1100°C and quenched to fabricate a hydratable mixture of CA. Then CaO-Al_2_O_3_-H_2_O was obtained by adding water to the calcium aluminates, and the hydrates were heated at 1100°C and quenched, resulting in CaO-Al_2_O_3_ clinker, which was ground to powder. To prepare the C3S/C12A7 mixture, the resultant C3S powder was mixed with C12A7 powder at proportions of 0, 5, 8, and 10 wt% of C12A7 (noted as C12A7-0, −5, −8, and −10, respectively).

### Initial setting time

We measured the initial setting time according to the recommendations specified in ISO 6876:2012. Each of the four powder groups was mixed with distilled water (DW) at a liquid-to-powder ratio of 0.4 mL/g and then inserted into Teflon molds of 1-mm thickness and 10-mm diameter. The initial setting time of the specimen (n=3) was measured using a Gilmore apparatus at 30-s intervals. Then, a 1/4-pound stainless steel indenter was applied vertically on to the horizontal surface of the specimen for 5 s. The initial setting time was determined as the time at which the indenter ceased to leave a distinct indentation on the surface of the specimen.

### Compressive strength test

Compressive strength was determined according to ISO 9917-1:2003. After the powder was mixed with DW, the resulting cement was inserted into Teflon molds of 6-mm thickness and 4-mm diameter and allowed to set at 37°C (n=10). After 1 or 7 days, compressive strength was measured using a universal testing machine (Z020, Zwick GmbH, Ulm, Germany) with a 0.5-N load cell at a crosshead speed of 1.0 mm/min. We calculated compressive strength with following formula: C=4P/D^2^, where C is the compressive strength (MPa), P is the maximum load force before fracture (N), and D is the diameter (mm) of the specimen.

### Alkalinity

Alkalinity was evaluated by measuring pH according to a previously published study.^11^ In brief, we prepared specimen (1-mm thickness and 5-mm diameter) and allowed to set completely. After setting, we inserted one tablet into 10 mL of deionized water. Then, the pH value was measured using a pH meter (Orion 3 Star; Thermo Fisher Scientific, Singapore).

### Primary culture of human dental pulp cells (hDPCs)

The experimental procedures with hDPCs of this study were approved by the institutional review board (IRB No: 2018-02-013). From the sectioned teeth, dental pulp tissue was obtained aseptically and rinsed with phosphate buffered saline (PBS; HyClone Laboratories Inc., Logan, UT, USA). The tissue was minced into small fragments in a 60-mm dish (Nunc, Roskilde, Denmark) and cultured in Minimum Essential Medium-α (MEM-α; HyClone Laboratories Inc.) containing 10% fetal bovine serum (FBS; Invitrogen, Carlsbad, CA, USA), 100 U/mL penicillin and 100 U/mL streptomycin (Invitrogen) at 37°C in 5% CO_2_. The cells between the third and fifth passages were used in this study.

### Cell viability test

After the powder was mixed with DW, the cement was allowed to set in disc shaped-paraffin wax molds (2 mm × 10 mm) for 24 h at 37°C. The cement was sterilized under ultraviolet light for 1 h. Then, the specimen was immersed in MEM-α containing 10% FBS at a ratio of 0.5 cm^2^/mL for 3 days to prepare the material extracts. The cells (2 × 10^4^/well) were inoculated in 24-well culture plates and incubated in culture medium. After 24 h, the cells were treated with 1 mL of the prepared extracts (n=4). The sample which contained no material extract was used as negative control, and C12A7-0 was considered as positive control. After 24 or 48 h, cell viability was measured by using the 3-(4,5-dimethylthiazol-2-yl)-2,5-diphenyltetrazolium bromide (MTT) assay. Briefly, MTT solution (0.05%, 0.2 mL) was added to each well, and the cells were incubated for 2 h. Then, 0.2 mL of dimethyl sulfoxide (Amresco, Solon, OH, USA) was inserted to each well. After the plates were shaken for about 5 min, the optical density was measured spectrophotometrically at 590 nm by using a microplate reader (SPECTROstarNano, BMG Labtech, Ortenberg, Germany).

### Measurements of the numbers of hDPCs

Each powder sample for each of four groups was mixed with DW at a liquid-to-powder ratio of 0.4 mL/g and allowed to set in a disc shaped-paraffin wax mold (1 mm × 5 mm) for 24 h at 37°C. Cells (1 × 10^5^) were inoculated on each specimen in the 24-well plates and cultured for 48 h in culture media (n=3). Then, the cells were stained with 4’,6-diamidino-2-phenylindole (DAPI) (Invitrogen) and scanned by a confocal laser scanning microscope (LSM 510 META, Carl Zeiss, Jena, Germany). Cell numbers were analyzed quantitatively from acquired images with an image analysis program (Image J ver. 1.37; National Institutes of Health, Bethesda, MD, USA).

### Statistical analysis

The results were statistically analyzed using one-way analysis of variance (ANOVA) and Tukey's tests to detect any significance (p<0.05). Prior to the analysis, the Kolmogorov-Smirnov test was used for determining normal distribution. The analyses were performed using SPSS software (SPSS ver. 12.0; SPSS Inc., Chicago, IL, USA).

## Results

### Characterization of C3S and C12A7

**Figure 1:**
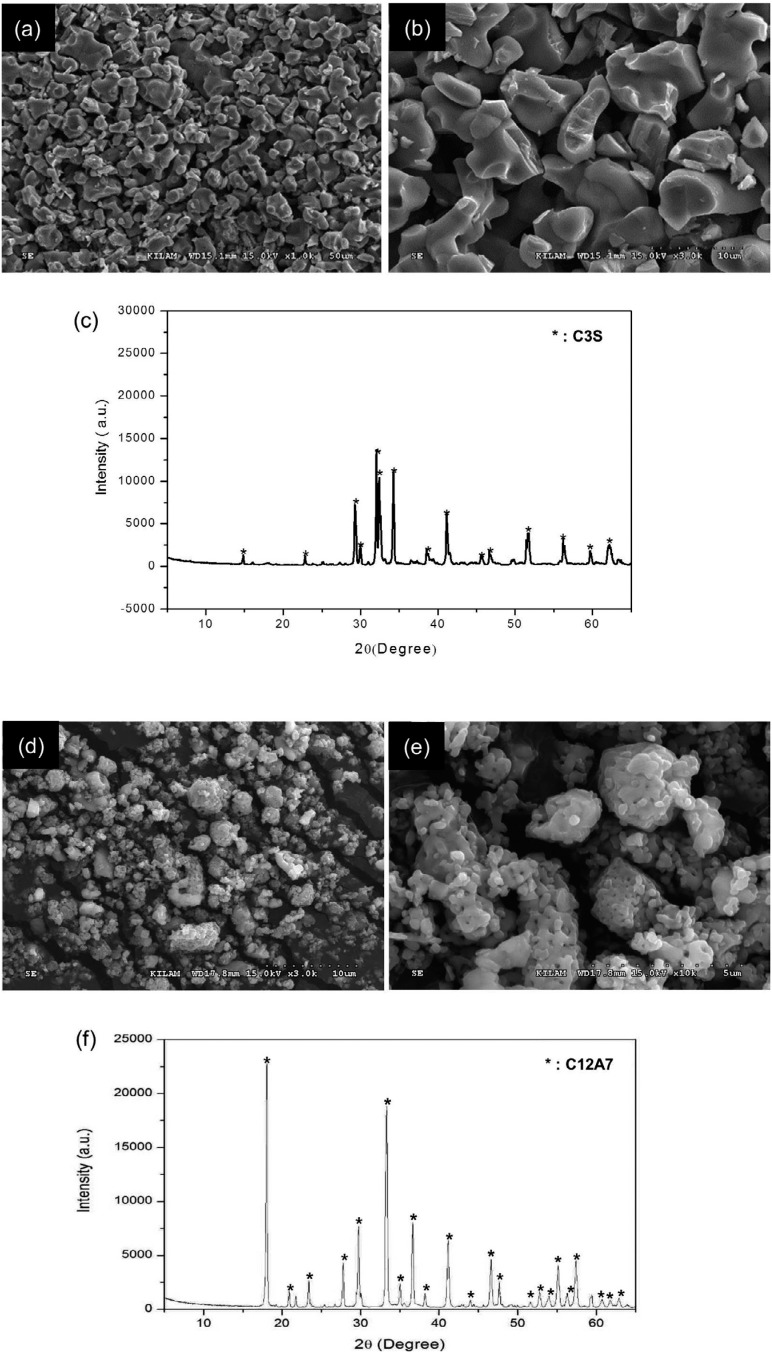
Characterization of prepared C3S and C12A7 powder; (A-B) Scanning electron microscope (SEM) images (x1000 and x3000, respectively) showed homogeneous compositions of particles in the C3S cement; (C) X-ray diffraction (XRD) characteristics of fabricated cement confirmed the powder as the C3S showing precisely identical peaks with that; (D-E) SEM images (x3000 and x10000, respectively) showed the characterization of the surface of C12A7 powder. (f) Fabricated powder was confirmed as C12A7 showing the same XRD peaks

The characteristics of prepared C3S and C12A7 were identified by SEM and XRD (Figure 1). The SEM images of fabricated C3S powders showed homogeneous composition of particles (Figure 1A and 1B). Phase analysis results by XRD indicated that the prepared powders were C3S and C12A7, respectively (Figure 1C and 1F). Quantitatively, the proportion of C3S was 100%, and particle size was less than 8 μm (Table 1).

**Table 1:** Characteristics of prepared cement powder. “d<n” is defined as the diameter at which n% of the sample's cumulative mass is comprised of particles with a diameter less than this value in the particle size distribution, and “dmax” is defined as the maximum diameter of particle size

Contents of evaluation	Results		Methods
Proportion of C3S (%)	100		XRD-rietveld refinement method
Particle size (μm)	d<10	0.84	Laser diffraction particle size analyzer
	d<50	1.92	
	d<90	3.89	
	Mean	2.17	
	dmax	8	

### Measurement of initial setting time, compressive strength, and alkalinity

**Figure 2:**
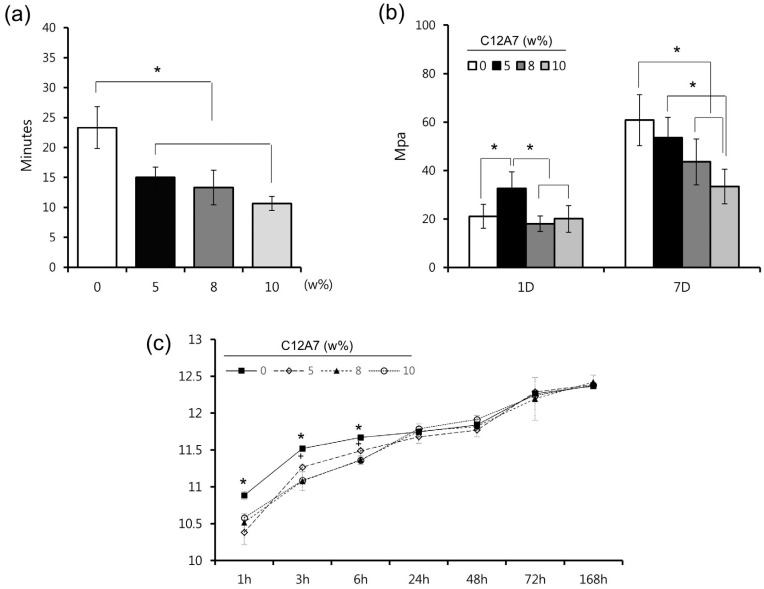
The physicochemical properties of C3S cement containing the various proportion of C12A7; (A) Initial setting time, (B) compressive strength, and (C) pH. statistical significance was determined at p<0.05

Initial setting time and compressive strength were measured to determine the physicochemical properties of cement with different ratios of C12A7. Regarding the initial setting time, there were significant differences between control (C12A7-0) and other samples (C12A7-5, −8, and −10) (p<0.05) (Figure 2A). As shown in Figure 2B, the compressive strength of C12A7-5 was significantly higher than that of other groups 1 day after the setting (p<0.05). However, there was no statistical difference between the other groups (p>0.05). After 7 days, the strength of C12A7-5 was similar to C12A7-0, whereas other groups showed lower strength compared to C12A7-0 (Figure 2B) (p>0.05). Regarding alkalinity, samples of all groups showed high pH values around 10 − 12 with an increasing pattern for 7 days. Although pH values of C12A7-0 were higher than other groups within 6 h (p<0.05), there were no significant differences after 1 day (Figure 2C). Furthermore, the C12A7-5 group showed higher pH value than C12A7-8 and −10 within 6 h (p<0.05).

### Measurement of viability and the number of hDPCs

**Figure 3:**
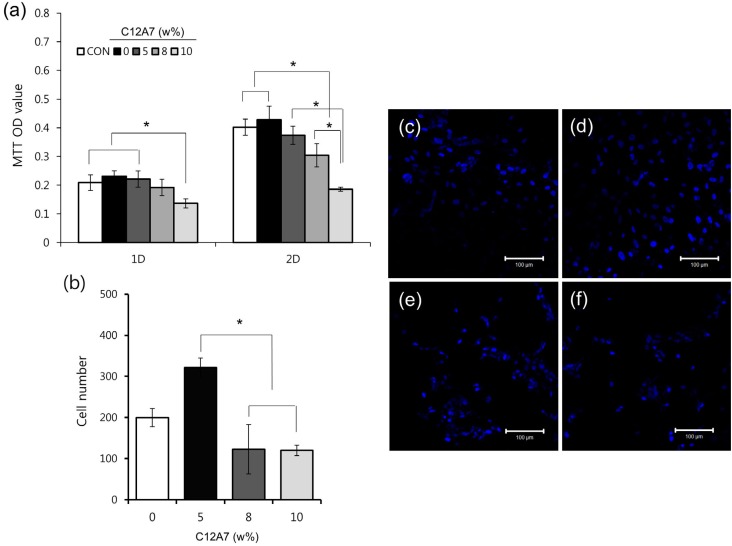
The cytocompatibility of C3S cement with the various proportions of C12A7; (A) The effects of C3S cement containing the various proportions of C12A7 on cell viability measured by the MTT assay; (B) The effects of C3S cement with the various proportions of C12A7 on the proliferation of hDPCs cultured on the specimens; (C-F) Representative confocal laser scanning microscopic images (x200) showing hDPCs (stained with DAPI) growing on the specimens (C12A7-0, 5, 8, and 10, respectively) after 48 h of culture. Statistical significance was determined at p<0.05

Viability and number of hDPCs were measured to investigate the cytocompatibiltiy of the cement. Although the cell viability of C12A7-containing cement decreased as the proportion of C12A7 increased, the cell viability of C12A7-5 was similar to that of both negative and positive control (C12A7-0) (p>0.05). Significantly lower cell viability than the control group in C12A7-10 was observed after 1 day and in C12A7-8 and −10 after 2 days (Figure 3A). Differences in cell number were also evaluated for C3S cement samples with various proportions of C12A7. While the cell numbers of C12A7-containing groups showed no statistical differences compared to the C12A7-0 group, the cell numbers of C12A7-5 were higher than those of C12A7-8 and −10 (p<0.05) (Figure 3B-F).

## Discussion

Portland cement acquired from natural materials can vary in composition and include impurities such as heavy metals of leachable lead and arsenic.^1^ According to several studies, commercially available MTAs may also contain heavy metals, although the amount released by MTAs is less than that of Portland cement.^12^ This contamination is of major concern in the development of cements that have high-purity CS, which leads to compositional stability and reliability, and the exclusion of heavy metal elements.^13^ Most new formulations of CS-based cements are currently based on C3S.^14^ C3S-based cement can be manufactured using pure raw materials, unlike Portland cement. C3S-based cement exhibits adequate physical properties and biologic performances, showing microstructure and hydration characteristics similar to those of Portland cement.^15^ In this study, we fabricated pure C3S cement with fine particles in the laboratory to exclude impurities and produce a stable composition of the base material (Figure 1A-C and Table 1). The processes of sintering, cooling and grinding were repeated to enhance the reaction rate of CaO-SiO_2_ by solid-state reaction, bringing about high proportions of C3S. The highly purified C3S cement we produced is expected to provide non-biased and stable results when used as a base material for evaluation of other additives in terms of effects on the base material.

Although C3S is largely responsible for initial setting and early strength of concrete used in the building industry, cement must have even more rapid setting ability for clinical applications. Several authors have shown that the addition of CA induces faster hydration reactions and improvements of the early mechanical strength of CS-based cement.^7^ ^,^ ^10^ C3A has been used as a representative additive in CS-based cement. However, the synthesis of non-blended C3A clinker is an energy-consuming process, because sintering must be repeated several times to avoid eutectic and peritectic reactions on the CaO-Al_2_O_3_ phase diagram. In contrast, C12A7 is more efficient to fabricate than C3A, due to its favorable sinterability. In addition, C12A7, known as mayenite, is one of the intermediary phases of the CaO-Al_2_O_3_ binary system and is also known to contribute to the first stage of strength development in aluminous cements like C3A.^16^ Therefore, C12A7 was selected for use in this study and was fabricated by sintering processes (Figure 1D-F).

Despite characteristics related to initial rapid hydration, high concentrations of CA can result in low final mechanical strength and negative effects on biocompatibility.^7^ Hence, we prepared various proportions of C12A7/C3S cement by mixing C12A7 with C3S cement to investigate the best proportions of C12A7 in C3S cement. In clinical contexts, MTA is surrounded by vital tissues like pulp and periodontal ligaments, as well as tissue fluids and blood. Faster initial setting and greater early strength can provide better initial support in bioactive circumstances. According to the previous report, the initial setting time of Portland cement was around 25 min,^17^ which is similar to our results of pure C3S (C12A7-0) around 23 min. On the other hand, commercially available products showed shortened initial setting time around 10-15 min.^17^ ^-^ ^19^ In C12A7-containing groups (C12A7-5, 8 and 10) of this study, the setting time was significantly shortened compared to pure the C3S group (C12A7-0), resulting in a value less than 15 min similar to that of commercial products (Figure 2A).^17^ ^-^ ^19^ In this respect, the addition of C12A7 in C3S cement showed significant benefit regarding the reduction of initial setting time. Furthermore, according to the results of the compressive strength test after 1 day, C12A7-5 showed higher early strength compared to C12A7-0. The compressive strength values obtained in this study were higher than those of MTA in another previous study.^20^ The results indicated that the addition of C12A7 might be beneficial for the early strength of the cement. Although the values of C12A7-8 and −10 decreased compared to control after 7 days, C12A7-5 showed no significant difference compared to the control group (Figure 2B). We also evaluated the pH of hydrated samples, since alkalinity is one of the most important characteristics of CS-based cement, as it is related to antimicrobial effect and dentin bridge formation near the pulp cells.^1^ ^,^ ^20^ ^,^ ^21^ The pH values increased during the period until 7 days, retaining high alkalinity at around 10 − 12, showing similar pattern and values with other previous studies using white MTA.^18^ ^,^ ^21^ Although the alkalinity of C12A7-0 was significantly higher in the early period within 6 h, there were no differences among all groups after 24 h (Figure 2C). Based on these findings from the initial setting time, compressive strength, and alkalinity test, we suggest that the addition of 5% C12A7 to C3S cement provide suitable benefit in terms of the clinical perspectives.

To evaluate the effect of C12A7 on the cytocompatibility of C3S-based cement in our study, we performed cell viability tests and measurements of cell number with hDPCs. Previously, several cell lines,^3^ ^,^ ^8^ ^,^ ^10^ were used for cell viability tests of CS-based cement. However, susceptibility to toxicity may vary between human- and animal-derived cells. Moreover, in clinics, as CS-based cement usually contacts exposed dental pulp when used as direct pulp capping materials, using the hDPCs can provide more exact informations.^22^ In our study, although cell viability decreased with increasing C12A7 concentration, the cell viability of C12A7-5 was similar to that of the control group after 1 and 2 days (p>0.05) (Figure 3A). Furthermore, the C12A7-5 group showed higher cell count than other groups (Figure 3B). In general, CS-based cement has proven to be biocompatible and to stimulate cell proliferation.^23^ It has been also reported that the dissolution of calcium and silicate ions from CS-based cements stimulates cell proliferation.^8^ ^,^ ^24^ However, the addition of CA can also exhibit negative effects on cytocompatibility in measures such as cell viability, attachment, and growth.^7^ It was also reported that, within the range of 10% of C3A in C3S, there was non-cytotoxic in the L929 cell.^10^ According to our results, we argue that adding 5% C12A7 to C3S cement does not negatively affect the cytocompatibility of hDPCs.

## Conclusions

In our study, we fabricated purified C3S cement and evaluated the effects of C12A7 when added to C3S cement, in order to identify a suitable proportion of this combination used in clinical contexts. Within the limitations of this study, we found that C3S-based cement with 5% C12A7 exhibited optimal characteristics, including faster initial setting time, improved compressive strength, and optimal alkalinity without adverse effects on cytocompatibility. Therefore, C3S-based cement containing 5% C12A7 could be used as a base material for the further study and development of new biomaterials for endodontic procedures.
